# The role of reperfusion injury in photodynamic therapy with 5-aminolaevulinic acid – a study on normal rat colon

**DOI:** 10.1038/sj.bjc.6600178

**Published:** 2002-03-18

**Authors:** A Curnow, S G Bown

**Affiliations:** Cornwall Dermatology Research Project, G14, Public Health Laboratories, Royal Cornwall Hospital, Treliske, Truro, Cornwall TR1 3LQ, UK; National Medical Laser Centre, Institute of Surgical Studies, Royal Free and University College London Medical School, 67–73 Riding House Street, London W1W 7EJ, UK

**Keywords:** 5-aminolaevulinic acid, light dose fractionation, photodynamic therapy, reperfusion injury

## Abstract

Reperfusion injury can occur when blood flow is restored after a transient period of ischaemia. The resulting cascade of reactive oxygen species damages tissue. This mechanism may contribute to the tissue damage produced by 5-aminolaevulinic acid-induced photodynamic therapy, if this treatment temporarily depletes oxygen in an area that is subsequently reoxygenated. This was investigated in the normal colon of female Wistar rats. All animals received 200 mg kg^−1^ 5-aminolaevulinic acid intravenously 2 h prior to 25 J (100 mW) of 628 nm light, which was delivered continuously or fractionated (5 J/150 second dark interval/20 J). Animals were recovered following surgery, killed 3 days later and the photodynamic therapy lesion measured macroscopically. The effects of reperfusion injury were removed from the experiments either through the administration of free radical scavengers (superoxide dismutase (10 mg kg^−1^) and catalase (7.5 mg kg^−1^) in combination) or allopurinol (an inhibitor of xanthine oxidase (50 mg kg^−1^)). Prior administration of the free radical scavengers and allopurinol abolished the macroscopic damage produced by 5-aminolaevulinic acid photodynamic therapy in this model, regardless of the light regime employed. As the specific inhibitor of xanthine oxidase (allopurinol) protected against photodynamic therapy damage, it is concluded that reperfusion injury is involved in the mechanism of photodynamic therapy in the rat colon.

*British Journal of Cancer* (2002) **86**, 989–992. DOI: 10.1038/sj/bjc/6600178 www.bjcancer.com

© 2002 Cancer Research UK

## 

Photodynamic Therapy (PDT) is a non-thermal technique, which can produce localised tissue necrosis, for the treatment of tumours and pre-malignant conditions in a range of organs. There is particular interest for treating pulmonary, gastrointestinal, skin and brain tumours as well as superficial bladder cancer (Stewart *et al*, 1998). PDT requires the systemic or topical administration of a photosensitiser, which is activated *in situ* by light of a specific wavelength to form cytotoxic species in the presence of molecular oxygen (Henderson and Dougherty, 1992).

PDT with the exogenous administration of 5-aminolaevulinic acid (ALA) is currently of interest, particularly for the treatment of superficial cutaneous tumours (Peng *et al*, 1997a). This compound enters the normal mammalian, tetrapyrrole biosynthetic pathway and is converted into a natural photosensitiser, protoporphyrin IX (PPIX) (Kennedy and Pottier, 1992). However, the effect produced by ALA-PDT is usually superficial, which often limits its clinical value (Regula *et al*, 1995; Barr *et al*, 1996). Experimentally, several techniques have been shown to enhance ALA-PDT. These include using a low light fluence rate (Robinson *et al*, 1998), ALA esters (Peng *et al*, 1996), iron chelators (Ortel *et al*, 1993; Curnow *et al*, 1998) and light dose fractionation (Messmann *et al*, 1995). In our studies, temporarily interrupting the light administration for 150 s increased the area of tissue necrosis by a factor of 3 (Curnow *et al*, 1999). The precise mechanism of this is not fully understood. It may be due to reoxygenation (Curnow *et al*, 2000) or relocalisation of the photosensitiser (Anholt and Moan, 1991) during the dark interval, but another possibility is reperfusion injury.

Reperfusion injury can occur as the blood supply is restored after a transient period of ischaemia. This happens as the enzyme, xanthine oxidase, becomes activated in a poorly oxygenated environment. Concomitantly, ATP is broken down, producing the purine substrates necessary for this enzyme to produce superoxide when oxygen is reintroduced (McCord, 1987). A schematic summary of this process is shown in Figure 1

**Figure 1:**
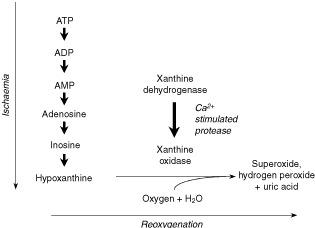
Schematic representation of the proposed mechanism for the ischaemia-induced production of reactive oxygen species as adapted from McCord (1985) with the kind permission of The New England Journal of Medicine.

. If reversible vasoconstriction occurs during the first fraction of a fractionated PDT treatment, this would result in an oxygen and thus energy compromised environment, making it conceivable that free radical damage could occur in this manner when the tissue is reperfused during the dark interval.

Superoxide is produced in most, if not all aerobic cells and the enzyme superoxide dismutase (SOD) is present intracellularly to remove it. Dismutation of superoxide produces hydrogen peroxide and the enzymes catalase (CAT) and glutathione peroxidase exist to remove this compound. Together, these enzymes protect cells against the reduced oxygen intermediates produced during normal aerobic metabolism. When superoxide and hydrogen peroxide production is excessive, however, such as following reperfusion injury, the protective enzymes are unable to cope, thus resulting in cell damage. Some of this damage may be mediated by the transition metal, iron, which can convert superoxide and hydrogen peroxide into aggressive oxidants such as the hydroxyl radical (by Haber-Weiss and Fenton-type reactions), which can then damage DNA, proteins and lipids, resulting in cell death. It is also possible that hydroxyl radicals can be produced (in a reaction independent of metal ions) from peroxynitrite (which itself can damage the cell membrane). Peroxynitrite can be produced from superoxide and nitric oxygen (which can also be toxic to cells) both of which can be increased when ischaemic tissues are reperfused (Gutteridge and Halliwell, 1990). The ultimate destructive effect of these reactive species is amplified many times by the cascade of free radical production they generate. Figure 2

**Figure 2:**
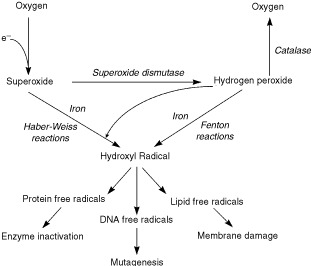
Summary of some of the reactions possible following reperfusion injury as adapted from Hammond *et al* (1985) with the kind permission of The Canadian Journal of Physiology and Pharmacology.

summarises some of these reactions, which are possible following reperfusion injury.

If SOD and CAT are present together in large quantities prior to ischaemia or at the start of reperfusion (through exogenous administration), they can produce significant protection against reperfusion injury (Gross *et al*, 1986; Jolly *et al*, 1984; Mittal *et al*, 1988). Allopurinol is a potent and highly specific inhibitor of the conversion of xanthine dehydrogenase to xanthine oxidase and can also be used to prevent reperfusion injury (Crowell *et al*, 1969; Paller *et al*, 1984).

This study investigates the contribution of reperfusion injury to the damage produced by continuous and fractionated PDT light regimes through the administration of free radical scavengers (SOD and CAT) and an enzyme inhibitor (allopurinol) to prevent any tissue damage caused by this mechanism. PDT is being developed for cancer therapy, but most of the studies designed to elucidate its mechanism of action and enhance the effect have been undertaken on normal tissues (Messmann *et al*, 1995) as experimental conditions are so much easier to control than in malignant tissue. This approach has led to many of the most important advances in establishing when it is safe and appropriate to use PDT in the treatment of human cancers (Bown, 1990). Reperfusion injury has not been studied previously in relation to PDT and although the response of normal and tumour tissue to PDT related reperfusion injury may not be exactly the same, it would be inappropriate to try and understand it in tumour tissue until the basic principles had been studied in normal tissue. For these reasons, these experiments were undertaken on normal rat colon, a model on which we have carried out much basic work on PDT over a period of many years.

## MATERIALS AND METHODS

### Chemicals

ALA powder (DUSA Pharmaceuticals, USA) was dissolved in physiological strength phosphate buffered saline (PBS) and given intravenously at a concentration of 200 mg ml^−1^ and a maximum volume of 0.2 ml. Superoxide dismutase and catalase (Sigma-Aldrich Co. Ltd., Poole, UK) were dissolved together in PBS and were administered intravenously at a concentration of 23.3 and 10 KU ml^−1^ respectively, maximum volume 0.2 ml. Allopurinol (Sigma-Aldrich Co. Ltd., Poole, UK) was dissolved in PBS and administered intravenously at a concentration of 50 mg ml^−1^ and a maximum volume of 0.2 ml.

### Animal model

Normal, female, Wistar rats (120–200 g) were used throughout and all procedures were conducted under licences granted by the UK Home Office in accordance with their regulations and the UKCCCR Guidelines (UKCCCR, 1998). The animals were anaesthetised for all parts of the procedure using inhaled halothane and analgesia was administered subcutaneously following surgery (buprenorphine hydrochloride).

### PDT studies

All animals were given 200 mg kg^−1^ ALA intravenously, 2 h prior to surgery. The colon was accessed for PDT via a laparotomy. The light source used was a 628 nm diode laser (Diomed Ltd., Cambridge, UK). A total energy of 25 J was delivered via a 200 μm plane cleaved optical fibre passed through the anti-mesenteric colon wall (approximately 1 cm distal to the caecum) so that it just touched the mucosa of the opposite side (area of contact=0.03 mm^2^). The power output of the optical fibre was kept at 100 mW. The rest of the abdominal viscera were shielded from forward light scatter by a piece of opaque paper positioned so that it did not touch the colon or affect its light distribution. This is a model that we have used many times in the past (Messmann *et al*, 1995). The light fluence rate where the fibre touches the tissue is high (320 W cm^2^) but no thermal effect was observed macroscopically in the light only control group at 3 days. As the light fluence rate falls rapidly with increasing distance along the colon wall from the fibre tip, measuring the area of the zone of necrosis in the wall of the colon is a convenient way of comparing the efficacy of PDT with different treatment parameters. The light dose was either delivered continuously or fractionated by a single interval of 150 s after one fifth of the energy dose had been delivered.

The inhibitory compounds (10 mg kg^−1^ SOD and 7.5 mg kg^−1^ CAT or 50 mg kg^−1^ allopurinol) were given intravenously either just before or immediately after illumination, the dosage and timing being chosen to mimic those of previous studies, where these compounds had been used successfully to prevent reperfusion injury (Crowell *et al*, 1969; Jolly *et al*, 1984; Paller *et al*, 1984; Gross *et al*, 1986; Mittal *et al*, 1988). Due to the short half-life of SOD in blood (6 min), the combination of SOD and CAT was administered as close to the beginning of the irradiation period as possible (Gross *et al*, 1986) whereas allopurinol was given 15 min prior to illumination. Animals were treated in groups of three with each type of inhibitory compound. Control groups (drug only, light only or both) received no radical scavengers or enzyme inhibitors. Animals were killed 3 days after light delivery and at post mortem, the area of PDT necrosis produced in the colon was determined macroscopically using an image analysis system. Some sections were examined histologically to confirm the macroscopic findings.

Statistical analysis of the area of necrosis between each treatment group and the appropriate control group was conducted using unpaired Student *t*-tests.

## RESULTS

At post mortem, the lesions seen in the colon were approximately elliptical when the colon was opened and laid flat. Histological analysis on representative specimens showed necrosis of the same extent as that measured macroscopically on the mucosa surface. The area of necrosis (mm^2^) produced by each of the treatment regimes is shown in Table 1

**Table 1:**
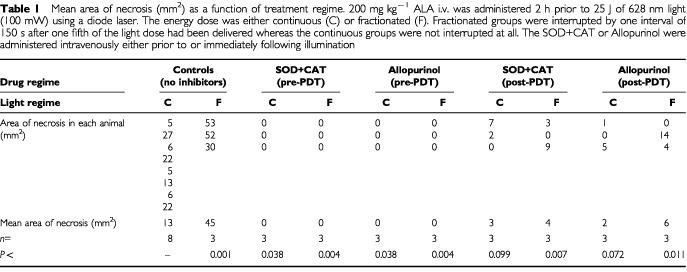
Mean area of necrosis (mm^2^) as a function of treatment regime. 200 mg kg^−1^ ALA i.v. was administered 2 h prior to 25 J of 628 nm light (100 mW) using a diode laser. The energy dose was either continuous (C) or fractionated (F). Fractionated groups were interrupted by one interval of 150 s after one fifth of the light dose had been delivered whereas the continuous groups were not interrupted at all. The SOD+CAT or Allopurinol were administered intravenously either prior to or immediately following illumination

. There was no effect in animals receiving either drug alone or light alone. Control groups receiving PDT (drug and light) but no scavengers or enzyme inhibitors produced areas of necrosis, with the fractionated group producing approximately three times the area of necrosis seen after continuous illumination. This finding is consistent with our previous work (Curnow *et al*, 1999), although the absolute areas were smaller than in the previous publication as it was necessary to use a 628 nm laser rather than the optimum wavelength of 635 nm. Use of the scavengers and enzyme inhibitor prior to irradiation completely inhibited the PDT effect. When these agents were given after illumination, the PDT effect was drastically reduced, although not completely eliminated.

## DISCUSSION

This study has shown that the administration of either the scavenging enzymes, SOD and CAT, or the xanthine oxidase inhibitor, allopurinol, prior to illumination, can prevent ALA-PDT from producing damage in the normal rat colon, whether the light dose is fractionated or continuous.

SOD and CAT remove harmful superoxide and hydrogen peroxide. These are known to be produced during reperfusion injury, but the effect is not specific. It is conceivable that cellular damage via singlet oxygen production would trigger a similar cascade of oxygen free radicals and reduced oxygen intermediates, particularly as the mitochondrion is reported to be a major site of ALA-PDT induced damage (Peng *et al*, 1997b). This would uncouple the electron transport chain ‘leaking’ further electrons to oxygen to form these reactive oxygen species. The disruption of cellular membranes has also been reported and once lysis of cellular compartments has been induced, the resultant free radical cascade would be cataclysmic for the cell.

The experiments with allopurinol are different. Allopurinol is known to be a specific inhibitor of the conversion of xanthine dehydrogenase into xanthine oxidase, which results in allopurinol being a specific inhibitor of reperfusion injury. The pre-administration of this compound would not be expected to have any effect on the production of reactive oxygen species or free radicals by any mechanism other than reperfusion injury. As the presence of this compound prior to light irradiation prevented the necrosis normally produced by ALA-PDT, it can be concluded that reperfusion injury must be playing some role in producing the necrosis in this animal model. It is known that if a tissue is not perfused, even for a period of time as short as 10 s (Roy and McCord, 1983), it is possible for xanthine dehydrogenase to be converted into xanthine oxidase. From previous studies monitoring oxygen levels during similar continuous and fractionated ALA-PDT treatments (Curnow *et al*, 2000), the tissue oxygen level can fall to zero at sites that subsequently become necrotic, even though there may be some temporary recovery of oxygen levels shortly after completion of illumination. This reoxygenation, which occurs shortly after the irradiation period finishes, may explain why the inhibitory compounds in the present study were not so effective when administered just after light irradiation rather than prior to illumination. Some re-oxygenation after light delivery would, of course, be essential for reperfusion injury to play a part in any PDT, with or without fractionation.

Our results suggest that reperfusion injury may play a part in producing PDT necrosis, at least with ALA. It could also explain the increased area of necrosis produced by fractionating the light as a result of reperfusion injury following reversal of temporary ischaemia in the treated area after the first light fraction as well as after completion of the final light fraction.
